# Incidence rates of the primary brain tumours in Georgia - a population-based study

**DOI:** 10.1186/1471-2377-14-29

**Published:** 2014-02-14

**Authors:** David Gigineishvili, Teimuraz Gigineishvili, Alexander Tsiskaridze, Roman Shakarishvili

**Affiliations:** 1 Department of Neurology & Neurosurgery, Faculty of Medicine, Javakhishvili Tbilisi State University, Tbilisi 0112, Georgia; 2 Sarajishvili Institute of Neurology, Tevdore Mgvdlis 13, 3rd floor, Tbilisi 0112, Georgia

**Keywords:** Epidemiology, Incidence, Primary brain tumours, Brain tumour registry, Meningioma, Glioblastoma

## Abstract

**Background:**

To determine the incidence rate and to describe other basic epidemiological data of primary brain tumours in a population-based study in Georgia, performed between March 2009 and March 2011.

**Methods:**

Active case ascertainment was used to identify brain tumour cases by searching neuroradiology scan reports and medical records from all participating medical institutions, covering almost 100% of the neurooncology patients in the country.

**Results:**

A total of 980 new cases were identified during the two-year period. For a population of almost 4.5 million, the overall annual incidence rate was 10.62 per 100,000 person-years, age-standardized to the year 2000 US population (ASR). Non-malignant tumours constituted about 65.5% of all tumours. Males accounted for 44% and females for 56% of the cases. Among classified tumours, age-standardized incidence rates by histology were highest for meningiomas (2.65/100,000), pituitary adenoma (1.23/100,000) and glioblastomas (0.51/100,000). ASR were higher among females than males for all primary brain tumours (10.35 vs. 9.48/100,000) as well as for main histology groups except for neuroepithelial, lymphomas and germ cell tumours.

**Conclusions:**

The annual incidence rate of all primary brain tumours in Georgia, though comparable with some European registry data, is low in comparison with the 2004–2005 Central Brain Tumor Registry of the United States (CBTRUS) database, which may reflect variations in reporting and methodology. The higher percentage of unclassified tumours (37.8%) probably also affects the discrepancies between our and CBTRUS findings. However, the most frequently reported tumour was meningioma with a significant predominance in females, which is consistent with CBTRUS data.

## Background

The determination of incident brain tumour cases is important in order to describe disease patterns and to identify the causes of the disease, and it is essential for the management, evaluation and planning of healthcare services for disease control.

Information about incidence rates of primary brain tumours in Georgia during last decades was only available from hospital-based pilot studies. According to these studies carried out in the Sarajishvili Institute of Neurology & Neurosurgery in 1996–2000, meningioma and glioblastoma were the most frequent diagnosed tumours and accounted for approximately 2/3 of all primary brain tumours [1]. At that time, new imaging techniques, which can enhance cancer detection rate, were established and situated only at hospital departments. In past decade, the situation changed radically. With some delay, advanced neuroimaging machines have been widely introduced in Georgia, and several state-of-art 1.5 tesla MRI units and multi-slice CT scanners are available both in clinical and ambulatory settings. Since imaging of the brain provides accurate information about brain structures, nowadays, a patient with a suspected brain tumour is routinely examined by an imaging diagnostic tool, which is a painless, noninvasive and fast medical test. In these circumstances, a review of CT and MRI results is a necessary step for obtaining accurate information about brain tumour incidence.

The present study used the database of a large population-based study performed in Georgia aiming to examine the incidence and the clinical and pathological features of primary malignant and non-malignant brain tumours. The first year results of the study have been reported recently [2]. We performed the analysis of two years data in order to increase the statistical power. In the absence of a national population-based brain cancer registry and, therefore, standard tumor registration procedures and training, cases are ascertained by an active search of neurosurgery hospital records and scan reports from CT and MRI units. This method allows for a precise estimation of the proportion of patients that are diagnosed only neuroradiologically and of those that undergo surgery with further histological verification.

## Methods

The cancer cases included in this study were patients diagnosed with primary brain tumour in Georgia between March 1st, 2009 and March 1st, 2011. All cases were coded following the International Classification of Diseases for Oncology, Third Edition (ICD-O-3). The information about the patients was gained from participating medical institutions: 15 different hospitals providing neurosurgical and neuroradiological services and numerous separate ambulatory-based CT and MRI units located in three large cities (Tbilisi, Kutaisi and Batumi). According to our estimates the participating medical facilities represent almost 100% of the neurooncological activity in the country. Ethical approval was obtained from the Tbilisi State University Medical Faculty ethics committee.

The active case ascertainment was used in order to capture all cases of newly diagnosed brain tumours within the study period. Relevant information (including socioeconomic, clinical, pathological, radiological and other details) was regularly collected by our representatives from the participating hospitals and neuroradiological units using a specially designed cancer case reporting form. Along with the written form, all collected data was kept in an electronic database. The cases were identified from: (1) medical records of all neurosurgical patients with a discharge cancer diagnosis, and (2) scan reports containing any suspicion of a brain tumour. Scan reports were further reviewed by a neurosurgeon to identify incident cases of primary brain tumours and to formulate a diagnosis for each selected case. If the description was limited, the tumor was qualified as ‘unclassified’. All final radiological cases were matched with the surgical cases to find possible duplicates and eliminate it in favour of the surgery report. If the diagnoses of the same patient differed between the radiological and the surgical database, the case was discussed with a neurosurgeon and the neurologist (DG) to obtain a single final diagnosis.

To ensure the completeness of the collected data, additional sources of information were used. The surgery database of the neurosurgical departments in Tbilisi hospitals and the large pathology database in Tbilisi were searched for cases coded with a brain cancer. For further verification, data obtained from clinical and radiological sources were checked against a histological database and in case of any disagreement over the diagnosis the diagnosis from pathology report was kept.

Selection criteria included all cases of intracranially located malignant or non-malignant tumours and excluded cases of recurrent brain tumour and extracranial tumour with invasion into cranium. Intracranial tumours that originated from the brain itself, meninges, cranial nerves, pituitary and pineal glands and craniopharyngeal duct were included in the study, according to the internationally recognized primary brain tumour standard definition [3]. The latest WHO 2007 histological classification of tumours of the central nervous system was used to classify cases into six main histology groups: neuroepithelial tumours, tumours of cranial and paracranial nerves, tumours of meninges, lymphomas and hematopoetic tumours, germ cell tumours and sellar region tumours [4].

The following information was selected and stored in the cancer case reporting form: demographics, diagnosis details (grade, behaviour, diagnosis confirmation method and date of diagnosis), treatment (surgery, radio- or chemotherapy), vital status and possible risk factor exposure. Behaviour code (0, 1, 3) was specified according to the ICD-O-3. Non–malignant primary brain tumors include those tumors with a benign behavior code of “0” or uncertain behavior code of “1”. The date of the first brain scan when cancer was detected was defined as the date of diagnosis. Besides tumour behaviour and grade three other variables were used to characterize a cancer type: histology group, specific histology and ICD-O-3 morphology code. Using these variables, we tried to further classify all un-formulated diagnoses as much as possible based on available data.

STATA and Microsoft Excel were used to calculate crude incidence rates, incidence rate ratio, frequency, arithmetic means of parameters and 95% confidence intervals (CIs) within the cohort and subgroups. Groups were compared by Student’s *t* test and the χ^2^ test for continuous variables and proportions, respectively. The denominator used for the analysis was the Georgian population in 2009 and in 2010, thus person-years at risk were calculated by summarizing the population data of both years. Age-standardization was performed based on five-year age groups across the whole age spectrum (total of 18 groups) and standardized to the US 2000 population in order to directly compare our incidence rates to those of the US Central Brain Tumor Registry (CBTRUS) since it contains the largest compilation of population–based data on the incidence of all primary brain tumours [5]. If a particular group consisted of less than 20 patients ASR and corresponding 95% CIs were not calculated. Calculating age-standardized rates (direct standardisation), standard errors, and 95% CIs of the standardized rate ratio (SRR) between groups selected by sex and histology were performed according to the method described by Boyle and Parkin [6].

## Results

### Basic characteristics

A total of 980 newly diagnosed cases of primary brain tumours were identified in the two-year period between March 2009 and March 2011. 44% of cases were male. According to the National Statistics Office of Georgia in 2009 there were 4.385 million inhabitants and4.436 million inhabitants in 2010 [7]. The mean age at diagnosis was 48.9 years (standard deviation [SD] 18.1 years). In the population below 20 years of age, only 83 cases were identified (8.5%). In 59.5% of the cases, the diagnoses were based on neuroradiological data; histological confirmation was received in 35.7%, and in the remaining 4.8% tumours were diagnosed based solely on clinical data (i.e., either the medical record did not contain a pathology report or pathology report was not available at the moment of data collection). Less than half of the patients (n = 404, 41.2%) underwent neurosurgical intervention. Some data sources (mostly scan reports) had incomplete demographic and/or clinical information, therefore several variables had missing values (as shown in Table 1); the numbers and percentages presented in text and tables are based on available data only.

**Table 1:** Percentage of missing values in several variables

**Variables**	**Total**	**Missing (%)**	**Unclassified (%)**
Case numbers	980	0	-
Age	977	0.3%	-
Sex	916	6.5%	-
Histology group	980	0	37.8%
Specific histology	973	0.7%	37.8%
ICD-O morphology code	980	0	Unspecified 40.7%
Tumor behaviour	444	54.5%	-
Tumor grade	377	61.5%	-

### Overall incidence of brain tumours

The crude incidence rate was 11.11 per 100,000 person-years, whereas the overall annual age-standardized incidence rate (ASR) per 100,000 person-years was 10.62 (Table 2), with similar rates in 2009 (10.25) and 2010 (10.99). Among specified neoplasms (n = 444), benign and borderline tumours accounted for 65.5% (ASR = 3.15) and malignant brain tumours for the remaining 34.5% (ASR = 1.67). The difference in ASRs between benign/borderline and malignant tumors was statistically significant (SRR = 1.88, 95% CI: 1.55; 2.29). The dominance of non-malignant over malignant tumours was observed in both years.

**Table 2:** Crude and age-standardized incidence rates of primary brain tumours by sex (per 100,000 person-years)

**Group**	**Specific histology**	**WHO histology code**	**N**	**%**	**IR**		**Females**				**Males**				
						**ASR (CIs)**	**N**	**%**	**IR**	**ASR (CIs)**	**N**	**%**	**IR**	**ASR (CIs)**	**SRR (CIs)**
Neuroepithelial			129	13.16	1.46	1.43 (1.18-1.68)	62	12.1	1.33	1.26 (0.94-1.58)	65	16.1	1.55	1.56 (1.18-1.95)	0.81 (0.57-1.15)
	Pilocytic astrocytoma	9421	6	0.61	0.07		2	0.4	0.04		4	1.0	0.09		
	Diffuse astrocytoma	9420, 9400	11	1.12	0.13		5	1.0	0.11		6	1.5	0.14		
	Anaplastic astrocytoma	9401	14	1.43	0.16		7	1.4	0.15		7	1.7	0.17		
	Glioblastoma	9440, 9441	48	4.89	0.54	0.51 (0.36-0.66)	22	4.3	0.47	0.42 (0.24-0.60)	26	6.4	0.62	0.62 (0.38-0.86)	0.68 (0.38-1.22)
	Other astrocytomas	9450	7	0.71	0.08		1	0.2			1	0.25	0.02		
	Oligodendroglioma	9451	5	0.51	0.06		4	0.8	0.08		3	0.7	0.07		
	Anaplastic oligodendroglioma	9391, 9392	4	0.41	0.04		2	0.4	0.04		3	0.7	0.07		
	Oligoastrocytoma	9424, 9425	2	0.2	0.02		9	1.7	0.19		8	2.0	0.19		
	Ependymoma	9382	17	1.73	0.19		3	0.6	0.06		1	0.25	0.02		
	Neuronal - glial	9470, 9471	7	0.71	0.08		5	1.0			1	0.25	0.02		
	Embryonal - Medulloblastoma	9505, 9506, 9493	8	0.81	0.09		2	0.4	0.04		5	1.2	0.12		
Tumours of cranial and spinal nerves	Neurinoma	9560	31	3.17	0.35	0.34 (0.22-0.46)	20	3.9	0.43	0.4 (0.22-0.58)	10	2.5	0.24	0.25 (0.09-0.40)	1.61 (0.77-3.37)
Tumours of meninges			284	28.98	3.22	2.97 (2.62-3.32)	173	33.7	3.73	3.33 (2.82-3.84)	96	23.7	2.29	2.20 (1.76-2.65)	1.51 (1.18-1.93)
	Meningioma	9530, 9531, 9532, 9533, 9534, 9537, 9539	254	25.91	2.88	2.65 (2.32-2.98)	158	30.8	3.41	3.02 (2.54-3.51)	83	20.5	1.98	1.91 (1.50-2.33)	1.58 (1.22-2.06)
	Mesenchymal - lipoma, haemangioma	8850, 9120, 9150, 9220, 9180	21	2.14	0.24		11	2.1	0.24		9	2.2	0.21		
	Haemangioblastoma	9161	9	0.92	0.10		4	0.8	0.08		4	1	0.09		
Lymphomas	Lymphoma	9590	3	0.3	0.03		1	0.2	0.02		2	0.5	0.04		
Germ cell	Germinoma	9064, 9080	4	0.41	0.04		1	0.2	0.02		3	0.7	0.07		
Tumours of the sellar region			130	13.26	1.47	1.40 (1.16-1.65)	76	14.8	1.64	1.57 (1.21-1.93)	41	10.1	0.98	0.93 (0.65-1.22)	1.69 (1.16-2.45)
	Pituitary adenoma	8272	115	11.73	1.30	1.23 (1.00-1.46	67	13.1	1.44	1.36 (1.04-1.69)	36	8.9	0.86	0.82 (0.55-1.09)	1.67 (1.12-2.48)
	Craniopharyngioma	9350	15	1.53	0.17		9	1.7	0.19		5	1.2	0.12		
Unclassified	Unclassified	8000	399	40.71			179	34.9			187	46.3			
	Total		980		11.11	10.62 (9.94-11.29)	512		11.05	10.35 (9.44-11.26)	404		9.64	9.47 (8.54- 10.41)	1.09 (0.96-1.25)

Note: N – case number; IR – crude incidence rate; ASR – age-standardized incidence rate; SRR - standardized rate ratio.

### Distribution and incidence rates by histology

**Figure 1:**
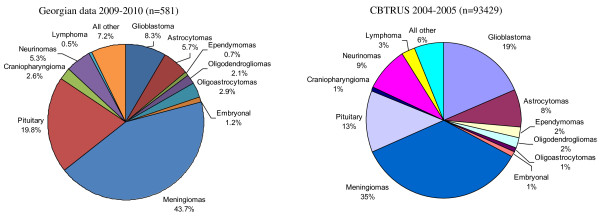
Distribution of primary brain tumours by histology (excluding unclassified cases).

**Figure 2:**
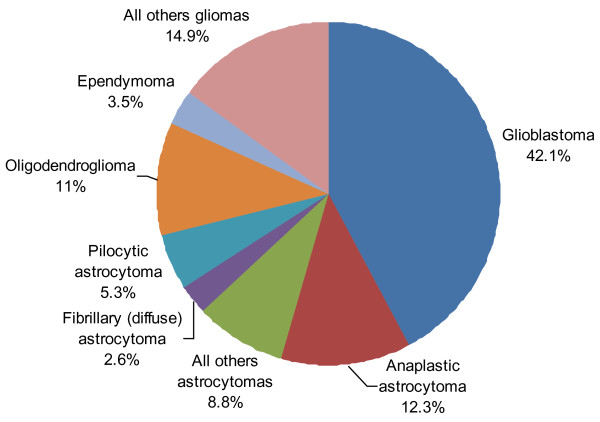
Distribution of all primary brain gliomas by specific histologies (n = 114).

The most frequent tumours by reported histology after excluding unclassified tumours were non-malignant meningiomas (n = 254, 43.7%), followed by tumours of sellar region (n = 130, 22.4%) (Figure 1). Gliomas, the most aggressive malignant brain tumours (astrocytic, oligodendroglial, oligoastrocytic and ependimal origin), represented 19.6% (n = 114) of all brain tumours. The distribution of glial tumours by specific histology is represented in Figure 2. Within this group, glioblastoma accounted for the majority of glioma.

Age-standardized and crude incidence rates among histology groups are shown in Table 2. The highest ASRs were observed for tumours of meninges (2.97 per 100,000 person-years), sellar region (1.40) and neuroepithelial tumours (1.43). Within specific histologies, ASRs were as follows: meningiomas (2.65), pituitary adenomas (1.23), glioblastomas (0.51) and neurinomas (0.34).

### Incidence rates and distribution by sex

The overall ASR was higher among females (10.35 versus 9.48 per 100,000 person-years) than males, but the difference was not statistically significant. Standardized incidence rates for non-malignant tumours were 1.5 times higher in females as compared to males (3.59 vs 2.36; SRR = 1.52, 95% CI: 1.19; 1.93), whereas for malignant tumours ASRs were 1.3 time higher in males than in females (1.92 vs 1.46 respectively; SRR = 1.32, 95% CI: 0.95; 1.82). Crude incidence rates by age are presented in Table 3.

**Table 3:** Crude incidence rates of brain tumours by age and sex in 2009–2010 (per 100,000 person-years)

	**Total**			**Female**			**Male**		
**Age range**	**Brain tumour cases**	**Population in 2009-2010**	**IR**	**Brain tumour cases**	**Population in 2009-2010**	**IR**	**Brain tumour cases**	**Population in 2009-2010**	**IR**
0 - 4	15	504,300	2.97	6	235,500	2.55	7	268,800	2.60
5 - 9	24	458,100	5.24	7	216,600	3.23	15	241,500	6.21
10 - 14	12	543,700	2.21	6	259,900	2.31	6	283,800	2.11
15 - 19	32	696,600	4.59	9	342,000	2.63	17	354,600	4.79
20 - 24	27	724,400	3.73	11	358,000	3.07	13	366,400	3.55
25 - 29	34	676,000	5.03	17	336,800	5.05	12	339,200	3.54
30 - 34	64	629,900	10.16	35	320,100	10.93	25	309,800	8.07
35 - 39	55	606,200	9.07	31	312,700	9.91	24	293,500	8.18
40 - 44	87	592,800	14.68	47	312,900	15.02	37	279,900	13.22
45 - 49	90	672,100	13.39	47	360,400	13.04	38	311,700	12.19
50 - 54	139	593,100	23.44	81	319,900	25.32	54	273,200	19.76
55 - 59	94	510,700	18.41	54	279,400	19.33	38	231,300	16.43
60 - 64	101	361,700	27.92	49	200,400	24.45	44	161,300	27.28
65 - 69	71	333,600	21.28	40	197,300	20.27	26	136,300	19.07
70 - 74	90	392,100	22.95	47	234,900	20.01	34	157,200	21.63
75 - 79	33	258,600	12.76	15	158,900	9.44	12	99,700	12.04
80 - 84	8	180,300	4.44	7	118,200	5.92	1	62,100	1.61
85+	1	87,600	1.14	1	68,200	1.47	0	19,400	0
Unspecified	3			2			1		
Total	980	8,821,800	11.11	512	4,632,100	11.05	404	4,189,700	9.64

For most of histology groups, primary brain tumours were more frequent in females as compared to males. The difference between ASRs was statistically significant for tumors of the meninges (3.33 vs 2.20) and of the sellar region (1.57 vs 0.93). In contrast, for specific tumours incidence rates of glioblastomas, embryonal tumours (medulloblastoma), lymphomas and germinomas were higher in males than in females, but the differences were not statistically significant (Table 2).

### Age distribution

**Figure 3:**
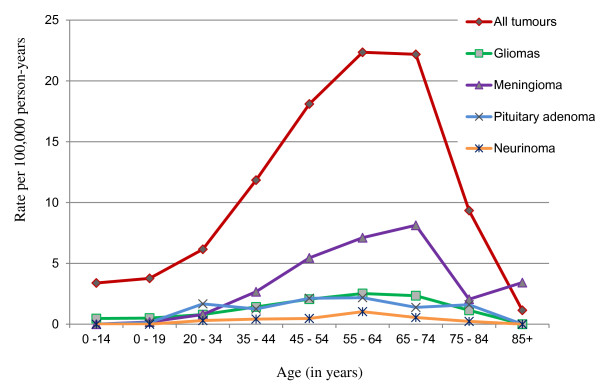
Age-specific crude incidence rates of primary brain tumours by histology.

Age-specific crude incidence rates for all tumours and selected histology groups are illustrated in Figure 3. Age-specific incidence rates were low for patients in late childhood and adolescence with a prominently increasing tendency in adulthood and a peak within the age range of 65–74 years.

## Discussion

### Comparison of incidence rates between registries

The present population-based study performed in the Caucasus region aimed to assess the incidence and clinical and pathological features of primary brain tumours using the WHO 2007 histological classification of tumours of the central nervous system. The observed overall ASR of primary brain tumours (10.62 per 100,000 person-years) is lower than the standardized rate of primary brain and CNS tumours diagnosed in 2004–2005 in the US and reported in 2009 by CBTRUS (18.16) and also lower than ASR reported by the Austrian Brain Tumour Registry (ABTR) in 2005 (18.1) [8,9]. Lower incidence rates compared with the CBTRUS database were also observed in our study for the main histology groups and specific histologies. However, in contrast to CBTRUS and ABTR data, recent publications from some European registries reported incidence rates of primary brain tumours similar to ours (8.5-14 per 100,000 person-years) [10-13].

The difference in the incidence rates may be due to several reasons. For example, a lower incidence rate in Georgia than in more affluent countries may reflect the low attention to subtle and sometimes obscure symptoms of brain tumours and as a consequence low healthcare utilization, complicated by insufficient or lack of health insurance coverage among the population. Additionally, CT and MRI imaging systems are concentrated only in large cities and therefore these diagnostic methods are too expensive and inaccessible for large parts of the rural population. A further, statistical explanation is that a high percentage of Georgian emigrants, who are looking for a job abroad (22.9% of the total population of over 4.4. million based on 2005 report of the United Nations Population Division and the World Bank), can artificially increase the denominator (i.e., ‘true’ population number is less than the official one) [14]. Finally, differences between countries in sociodemographic characteristics and environmental factors (so-called geographic variation factor), which may be associated with brain tumour risk, should be taken into account when interpreting ASRs of different countries.

### Direct comparison to CBTRUS statistical report

The comparison of tumour distribution patterns by behaviour and histology showed a high comparability of the rates in our study with the CBTRUS statistics, in particular the predominance of non-malignant over malignant tumours, with the most common histology being meningioma, and glioblastoma representing the most frequent type of gliomas. However, some slight differences can be found in distribution of specific tumour types according to their frequency as shown in Figure 1.

For the majority of tumours, incidence rates were higher among females than males, but for specific tumours the strongest differences by sex were observed for meningiomas and pituitary adenomas (common in females) and for glioblastoma and germ cell tumours (common in males). These results are concordant with those in the CBTRUS and ABTR reports, which may indicate the validity of our data. Moreover, age-specific rate curves for brain tumours among the registries were nearly identical with a prominent increase of the incidence rate after approximately 30 years of age and a decline in those over 75 years old. The drop in incidence is evident in our and ABTR data, but less pronounced in the CBTRUS report.

### Study limitations and next steps for improving validity of the data

Our study has limitations caused by the relatively low rate of histologically confirmed tumours (36%). In contrast, histological verification rate is 69% in CBTRUS report and 80.9% in the ABTR data. In other studies, however, pathological confirmation index varied widely from 35% to 59% [11,15,16]. The percentage of histologically verified cases is one of the registry reliability indicators and efforts should be directed towards improving this value, which is less likely to reflect bias in reporting cancer cases (the index in our study is persistently low in both years – 38% and 33.5%, respectively). There is some evidence in the literature, however, that because non-invasive neuroimaging techniques improve and become an important diagnostic tool today, the proportion of radiologically diagnosed tumor cases has increased, especially among older patients. Indeed, 41% of the histologically unverified tumours in the population-based cancer registry of Spanish province Girona were explained by easy access to sophisticated imaging tools [15]. We suggest that a limited neurosurgical activity in elderly patients, among whom nonsurgical treatment options are usually unavailable (in particular for patients in rural areas), contributes to higher number of neuroradiologically confirmed diagnoses. This is mostly also due to delays in diagnosis leading to presentation with advanced disease. All these factors predicted the relatively high proportion of unclassified tumours in our study. Indeed, our data confirm that radiologically defined cases constitute about 90% (n = 362) of unclassified group. The peak of purely radiologically identified cases with no further classification was observed in the age group 50–54 years and the percentage is persistently high up to 75 years of age. Analysing the causes of absence of histological verification among cases with suspected brain tumour is out of the scope of this study, but the following factors can be discussed. In addition to the above mentioned observation, cultural, educational and economical factors may predispose to a low level of activity among suspected patients, who are not motivated to visit a specialized neurooncology center. Also, an existing cancer program and the activity of medical services are under development and probably do not meet the existing demands. Much effort should be put into identification, registration and further management of neurooncology patients in accordance with current western standards. In this respect, establishing a fully-authorized brain cancer registry will introduce mandatory standards for registration processes, which minimize biases and incompleteness in registration practices. For example, technical and institutional barriers did not allow us to get access to the Georgian National Registry Bureau for searching death certificates. Although the missing cases (if any) most likely would not have affected the incidence rates in subgroups, establishing a centralized brain tumour registry will help to overcome any institutional barriers for getting relevant information.

## Conclusion

Despite the above mentioned limitations, our data show the brain cancer diagnosing procedure quality in a country with limited economical resources and consequences of political reorientation. In this respect, regularly reported data from a brain cancer registry will help to focus on diagnostic and missing data issues for improving the validity of the data. Additionally, population-based epidemiological data provide important information for the development of proper national healthcare strategies in relation to neuro-oncological patients. Regardless of the quality of currently available data, the current report is the only opportunity to assess the impact of cancer in the community and cancer registration should be regarded as a priority in the nationwide health policy.

## Abbreviations

ABTR: Austrian brain tumour registry; ASR: Age-standardized incidence rate; CBTRUS: Central brain tumor registry of the United States; CI: Confidence interval; CT: Computer tomography; IR: Incidence rate; MRI: Magnetic resonance imaging; SRR: Standardized rate ratio.

## Competing interests

Authors declare that they have no competing interests.

## Authors’ contributions

DG was involved in study concept/study design, data acquisition, data analysis/interpretation, statistical analysis, manuscript drafting, literature research, and all critical revisions of the manuscript. TG performed data acquisition, data analysis/interpretation and literature research. AT was involved in study concept/study design, and edited the manuscript. RS was involved in study concept/study design, data analysis/interpretation and edited the manuscript. All authors read and approved the final manuscript.

## Pre-publication history

The pre-publication history for this paper can be accessed here:

http://www.biomedcentral.com/1471-2377/14/29/prepub
